# The Relevance of G-Quadruplexes in Gene Promoters and the First Introns Associated with Transcriptional Regulation in Breast Cancer

**DOI:** 10.3390/ijms26146874

**Published:** 2025-07-17

**Authors:** Huiling Shu, Ke Xiao, Wenyong Zhu, Rongxin Zhang, Tiantong Tao, Xiao Sun

**Affiliations:** State Key Laboratory of Digital Medical Engineering, School of Biological Science and Medical Engineering, Southeast University, Nanjing 211189, China

**Keywords:** G-quadruplexes, the first intron, transcriptional control, transcription factor binding, breast cancer

## Abstract

The role of G-quadruplexes (G4s) in gene regulation has been widely documented, especially in gene promoters. However, the transcriptional mechanisms involving G4s in other regulatory regions remain largely unexplored. In this study, we integrated the G4-DNA data derived from 22 breast cancer patient-derived tumor xenograft (PDTX) models and MCF7 cell line as potential breast cancer-associated G4s (BC-G4s). Genome-wide analysis showed that BC-G4s are more prevalent in gene promoters and the first introns. The genes accommodating promoter or intronic BC-G4s show significantly higher transcriptional output than their non-G4 counterparts. The biased distribution of BC-G4s in close proximity to the transcription start site (TSS) is associated with an enrichment of transcription factor (TF) interactions. A significant negative correlation was detected between the G4–TF interactions within the first introns and their cognate promoters. These different interactions are complementary rather than redundant. Furthermore, the differentially expressed genes (DEGs) harboring promoter and first intron BC-G4s are significantly enriched in the cell cycle pathway. Notably, promoter BC-G4s of DEGs could be a central hub for TF–TF co-occurrence. Our analysis also revealed that G4-related single nucleotide variants (SNVs) affect the stability of G4 structures and the transcription of disease-related genes. Collectively, our results shed light on how BC-G4s within promoters and first introns regulate gene expression and reinforce the critical role of G4s and G4-related genes in breast cancer-associated processes.

## 1. Introduction

The DNA G-quadruplexes (G4s) are non-canonical four-stranded structures formed in guanine-rich regions via a union of four G-tracts [1]. The putative G4-forming sequences (pG4s) are prevalent in functionally important genomic regions, especially around the transcription start sites (TSSs) in the human genome [2,3]. A typical G4 motif is represented as G_x_N_1–7_G_x_N_1–7_G_x_N_1–7_G_x_N_1–7_ (where x ≥ 3 and N can be any base) [4]. Computational analyses demonstrate the presence of over 700,000 regions with the potential to fold into G4 structures in the human genome [5]. A number of studies have shown that G4s are implicated in various essential cellular processes including transcriptional regulation, chromatin remodeling, alternative splicing, and genome maintenance [6,7,8,9,10,11].

The impact of G4 structures on transcription activity has been widely discussed. Numerous bioinformatic studies have supported the idea that G4s are predominantly present at gene promoters, especially in oncogenes [12,13,14]. Accumulating evidence has revealed that endogenous G4s are enriched in active promoters which correlates with elevated transcription [15,16]. A comprehensive analysis of transcription factor (TF) binding sites and G4 structures emphasized G4s functioning as binding hubs for multiple TFs [17]. Consequently, such enhanced gene transcription is probably associated with the recruitment of increased numbers of TFs at promoter G4s [15]. Biochemical tests further verified that several TFs exhibit high-affinity binding to G4s in vitro including SP1, MAZ, and PARP-1 [18,19]. In contrast to the great interest in promoter G4s, G4s located downstream of the TSS, especially within introns, are less studied. It has been reported that first introns are the longest and the most conserved compared with other downstream introns [20,21,22]. In addition, first introns contain more regulatory elements and active epigenetic marks linked to the level of gene expression [21,23]. Examination of sequences downstream of the TSSs demonstrated that G-rich elements are concentrated in the first introns [24]. These observations lead us to hypothesize that G4s formed by G-rich intron 1 elements may serve as structural targets for modulating gene expression, hence it is necessary to dig into the mechanism that takes advantage of these structures.

Studies have shown that single nucleotide variants (SNVs) could also affect gene expression when located within transcription regulatory elements [8]. Since the majority of these SNVs are found in non-coding regions, it is still a challenge to elucidate the impact of SNVs on molecular function and disease [25,26]. Recent works highlighted a correlation between disease risk and the alteration of G4s caused by SNVs, which may be attributed to the change in gene expression related to the transformation as well as the gains/losses of G4 structures [8,27,28]. Thus, the connection between G4-related SNVs and the transcription of disease-associated genes in a specific cancer type deserves to be explored thoroughly.

Based on the significant role of G4s in multiple biological processes, G4 structures have been considered as a potential therapeutic tool against tumor cells [5]. An integrative analysis of differentially enriched G4 regions in breast cancer patient-derived tumor samples unveiled intratumor heterogeneity, thus contributing to breast cancer stratification and precision medicine for cancer treatment [29]. According to the GLOBOCAN cancer statistics for the year 2022, breast cancer has become the most frequently diagnosed cancer in 157 countries and it also ranked the leading cause of deaths in 112 countries [30]. Although a recent study focused on the different G4 patterns in distinct breast cancer subtypes [29], the overall characteristics of G4s in breast cancer, especially those in non-coding regulatory regions other than promoters, remain largely unexplored.

**Figure 1:**
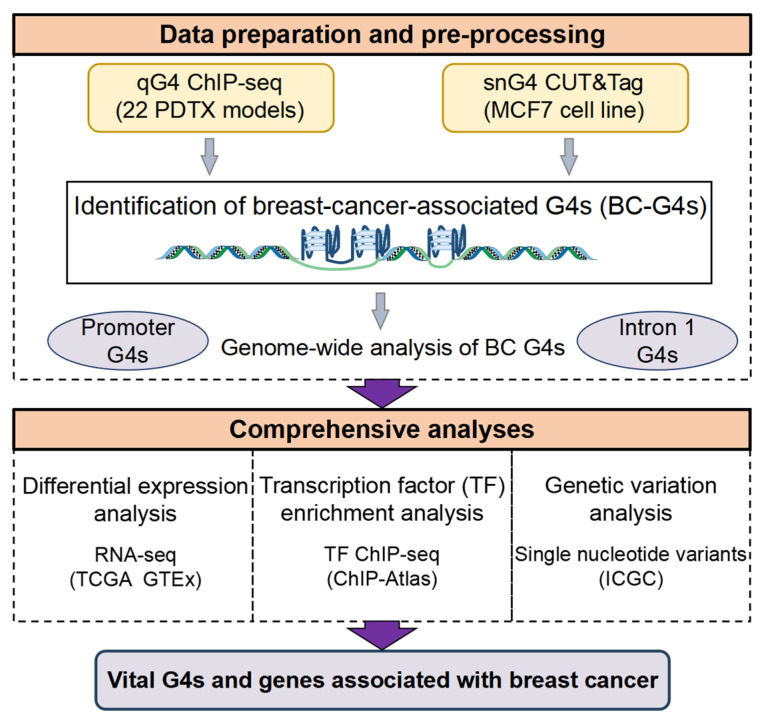
Bioinformatics pipeline towards the identification of vital G4s and G4-related genes associated with breast cancer. We first integrated G4 data from 22 breast cancer PDTX models and MCF7 cell line to identify BC-G4s in the human genome. The importance of G4s in gene promoters and the first introns was then demonstrated through genome-wide analysis of BC-G4s. The crucial BC-G4s and G4-related genes were characterized according to differential expression analysis, TF enrichment patterns and genetic variation analysis.

In this study, we first identified all candidate G4s associated with breast cancer (BC-G4s; see definition below) and investigated their genomic distribution patterns. We explored the relationship between BC-G4s and gene expression, focusing on the genes harboring BC-G4s in promoters and the first introns. Next, pathway enrichment patterns were analyzed on the differentially expressed genes (DEGs) with promoter and first intron BC-G4s. To elucidate the impact of BC-G4s on gene transcription, we systematically investigated TF interactions with BC-G4s in both promoters and the first introns. Additionally, we conducted TF enrichment analysis on promoter BC-G4s of DEGs and further explored the potential biological mechanisms underlying the TF network. Finally, we examined the alteration of BC-G4s caused by SNVs, as well as their influence on key genes related to breast cancer biology. Taken together, we discovered vital BC-G4s and G4-related genes which play a crucial role in breast cancer progression through in-depth analyses of breast cancer G4 data (Figure 1).

## 2. Results

### 2.1. BC-G4s Are Enriched in Gene Promoters and the First Introns

To comprehensively explore the roles of G4s in breast cancer development and progression, we mapped all candidate G4 structures correlated with breast tumorigenesis. We collected the in vivo G4 data from the public high-throughput experiments in breast cancer including 22 breast cancer patient-derived tumor xenograft (PDTX) models (27 biological samples) and the MCF7 breast adenocarcinoma cell line [29,31]. For each PDTX sample, peaks were considered as high-confident G4 regions if confirmed in two out of four technical replicates. These G4 sites were then merged to generate a single PDTX G4 dataset. Since approximately 78.6% of MCF7 G4s overlap with PDTX G4s (Supplementary Figure S1A), we finally merged them into a combined breast cancer-associated G4 dataset (hereafter called BC-G4s) for further analysis.

**Figure 2:**
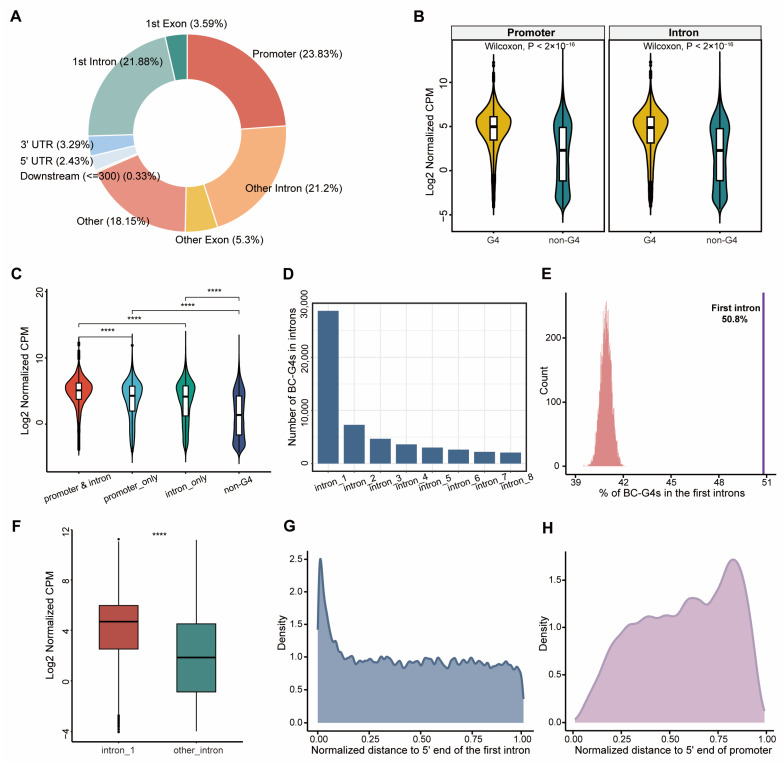
Genome-wide analysis of BC-G4s. (**A**) Distribution patterns of BC-G4s in the human genome. (**B**) Violin plots show the expression levels of genes harboring promoter or intronic BC-G4s compared with those without G4 regions. The yellow violin plot stands for the G4 group, whereas the green plot represents the non-G4 group. (**C**) Violin plots show the differences among the expression levels of the four gene categories. The red, light blue, green and dark blue violin plots signify the genes with both promoter and intronic BC-G4s, genes with only promoter G4, genes with only intronic G4s and genes without these G4 regions, respectively. (**D**) Distribution patterns of BC-G4s in intronic regions (from intron 1 to intron 8). (**E**) Histograms depict the proportions of BC-G4s in the first introns estimated during 10,000 random iterations. The actual proportion of BC-G4s in the first introns (50.8%) is marked by a purple line in the right panel, while the proportions of BC-G4s from the 10,000 permutations are shown as histograms in the left panel. (**F**) Boxplots show the expression of genes with BC-G4s in distinct introns. The dark red and green boxes indicate the genes accommodating BC-G4s in the first introns or the other introns, respectively. (**G**) Density plot illustrates the normalized distance of BC-G4s to 5′ end of the first intron. (**H**) Density plot illustrates the normalized distance of BC-G4s to 5′ end of the promoter. **** *p* < 0.0001, Wilcoxon two-sided test.

The genomic feature distributions of BC-G4s were first evaluated. Notably, BC-G4s are highly abundant in promoters and introns, especially in the first introns (Figure 2A). Motivated by the consideration that non-canonical DNA structures affect gene expression, we assessed whether the existence of BC-G4s in gene promoters and introns was coupled to the gene expression level. To this end, we systematically overlapped gene promoter and intronic regions with BC-G4s. We used counts per million (CPM) values normalized with the trimmed mean of M (TMM) values method derived from TCGA breast cancer raw count data to analyze expression profiles. Consistent with our hypothesis, the genes harboring promoter or intronic BC-G4s show significantly high expression when compared to those without such G4 regions (Figure 2B; Wilcoxon two-sided test, *p*-value < 2 × 10^−16^). To exclude the mutual influence of promoter and intronic BC-G4s on gene expression, we further divided genes into four categories: genes with both promoter and intronic G4s, genes with only promoter G4s, genes with only intronic G4s and genes without these G4 regions. Planned comparisons demonstrated that the expression level of the genes harboring both promoter and intronic G4s is significantly increased relative to the other groups (Figure 2C; Wilcoxon two-sided test, *p*-value < 2.22 × 10^−16^).

In particular, we investigated the presence of BC-G4s from intron 1 to intron 8 since the average gene transcript possesses approximately 7.6 introns. We found that BC-G4s are more prevalent in the first introns in comparison to the others, with a gradual decrease in the numbers of BC-G4s overlapping the succeeding introns (Figure 2D). Given that first introns are typically longer than other downstream introns [32], we probed whether the large proportion of BC-G4s in the first introns was attributed to the functional properties of the intron sites or simply an artifact of their long length. By conducting a permutation test randomizing the positions of 20,589 intronic BC-G4s across all intronic regions, we discovered that the observed proportion (50.8%) was not replicated in any of the 10,000 permutations, strongly eliminating the possibility that the proportion of BC-G4s in the first introns is solely a by-product of their long length (*p*-value << 0.0001, Figure 2E). Remarkably, the genes with BC-G4s in the first introns show substantially enhanced expression as compared to those with G4s in other introns (Figure 2F and Supplementary Figure S1B; Wilcoxon two-sided test, *p*-value = 1.7 × 10^−6^). Intriguingly, the distribution of BC-G4s has a bias towards the 5′ end of the first introns and 3′ end of promoters, indicating highly abundance in the vicinity of the TSSs (Figure 2G,H and Supplementary Figure S1C). In general, these results underscore the importance of BC-G4s in promoters and the first introns to gene expression in breast cancer.

### 2.2. The Distribution of BC-G4s in Up-Regulated Genes Is Biased Toward the TSSs

**Figure 3:**
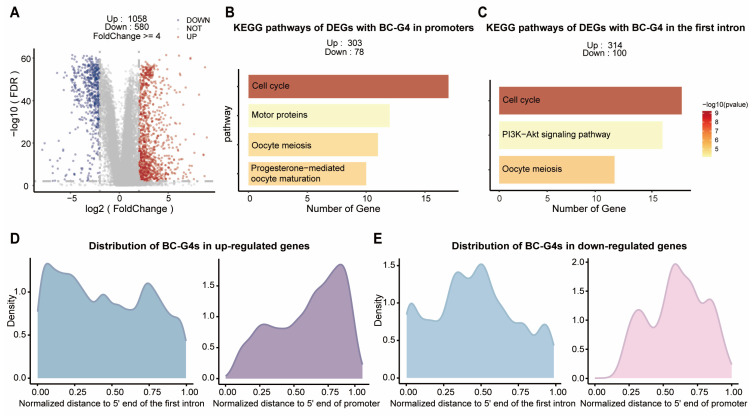
Characteristics of BC-G4s in the promoter and the first intron of the differentially expressed genes (DEGs). (**A**) The volcano plot depicts DEGs between tumor and NAT groups in breast cancer. The up-regulated and down-regulated genes are represented with red and blue colors, respectively. (**B**,**C**) describe the KEGG pathway enrichment results of DEGs with BC-G4s in gene promoters and the first introns, respectively. DEGs accommodating BC-G4s in promoters and the first introns are both remarkably enriched in cell cycle pathway. (**D**,**E**) show the distributions of the normalized distance of BC-G4s to 5′ end of the first intron and promoter in up-regulated and down-regulated genes, respectively.

To gain insight into the regulatory functions of BC-G4s, we focused on the promoter and first intron BC-G4s in the differentially expressed genes (DEGs). We integrated TCGA and GTEx raw count data processed using an identical pipeline to generate a transcriptomic profile comprising 1119 tumors, 113 tumor-adjacent normal (NAT) samples in breast cancer, and 92 normal samples in breast tissue [33,34]. We employed a stringent normalization method to remove unwanted variation across batches, enabling direct comparison of runs performed in different laboratories at different times (Supplementary Figure S2C) [35]. In total, 1058 up-regulated and 580 down-regulated genes were identified in tumors relative to NAT samples (Figure 3A). Among these DEGs, 303 up-regulated and 78 down-regulated genes contain promoter BC-G4s while 314 up-regulated and 100 down-regulated genes harbor BC-G4s in the first introns. Taken together, BC-G4s are prominently present in the promoters and first introns of up-regulated DEGs (Fisher’s exact test *p*-value = 8.992 × 10^−13^, *p*-value = 2.022 × 10^−8^ for promoter and the first intron, respectively).

To further explore the biological implications, we compared Kyoto Encyclopedia of Genes and Genomes (KEGG) pathway enrichment patterns between DEGs with BC-G4s in gene promoters and the first introns (Figure 3B,C). Interestingly, both groups are markedly enriched in cell cycle pathway compared to the DEGs lacking BC-G4s which indicates that BC-G4s may be an integral part in the regulation of cell cycle in breast cancer (Figure 3B,C and Supplementary Figure S2D). In addition, BC-G4s in the up-regulated genes are preferentially found in the 5′ end of the first introns and 3′ end of promoters, a pattern that is not observed in the down-regulated genes (Figure 3D,E and Supplementary Figure S2E). The proximity of BC-G4s to the TSSs in the up-regulated genes further underscores a pivotal role of G4 structures in the regulation of gene expression.

### 2.3. TF Binding to BC-G4s in the First Introns Compensates for G4–TF Interactions in the Cognate Promoters

**Figure 4:**
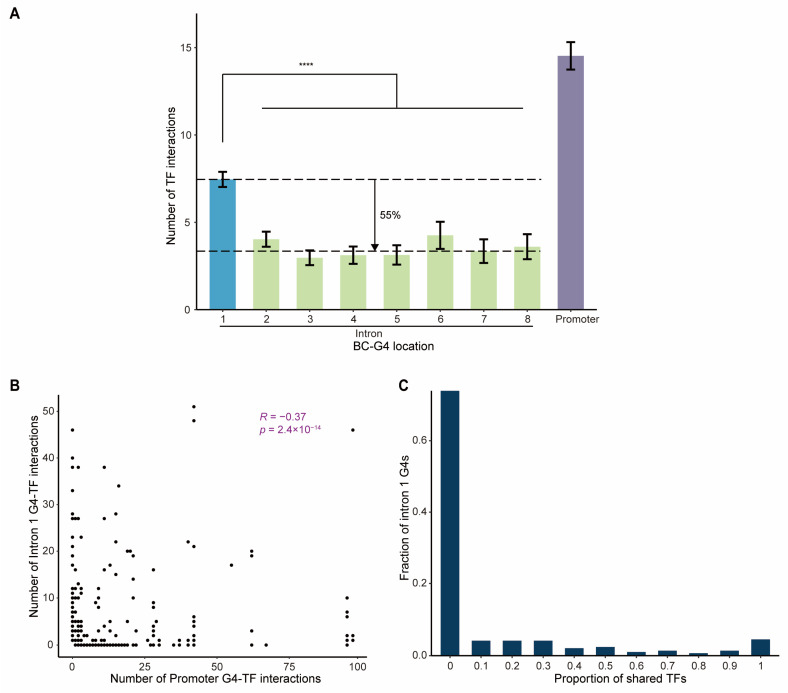
Relationship between promoter and first intron G4–TF interactions. (**A**) TF binding to intronic and promoter BC-G4s. The average number (±SEM) of TFs that interact with BC-G4s is plotted. The blue, green and purple color bars represent BC-G4s located in the first introns, other introns, and promoters, respectively. (**B**) Spearman correlation between the number of promoter and the first intron G4–TF interactions. (**C**) The barplot shows the distribution of the proportion of TFs that bind both promoter and first intron G4s, relative to the total TFs bound by first intron G4s, on a gene-by-gene basis. **** *p* < 0.0001, Mann–Whitney U test.

The fact that BC-G4s are highly abundant near the TSSs prompted us to further assess their potential role in transcriptional regulation. We first evaluated the frequency of TF interactions with BC-G4s in promoters and introns of DEGs using ChIP-seq data for 148 TFs enriched in G4s. The result reflected that BC-G4s in the first intron of DEGs bind over twice as many TFs as BC-G4s in downstream introns (Figure 4A; Mann–Whitney U test, *p*-value = 1.376 × 10^−15^). This supports the preceding conclusion that BC-G4s in the first introns have a greater impact on gene regulation than those in other introns. Considering that the first introns exhibit a higher density of active transcriptional regulatory signals [21], we speculated that BC-G4s in the first introns harbor more TF binding events due to functionality of the first intron sites and the proximity to the TSS.

The frequent binding of TFs to BC-G4s in both promoters and first introns hinted inner relationship between these two groups of BC-G4s. Moreover, a study of *Caenorhabditis elegans* suggested that TFs binding to first introns are largely distinct from those binding to promoters, and the different interactions are complementary rather than redundant [22]. Therefore, we tested this hypothesis for the BC-G4s by comparing the number of G4–TF interactions between promoters and first introns for each gene. As expected, we detected a significant negative correlation between promoter and the first intron G4–TF interactions, implying that TF binding to both regions is mutually complementary to a certain extent in breast cancer (Figure 4B; Spearman’s correlation coefficient R = −0.37, *p*-value = 2.4 × 10^−14^).

Furthermore, we hypothesized that G4–TF interactions in the first introns may contribute to transcriptional regulation in two alternative ways. On one hand, BC-G4s in the first introns could share some identical TFs with those in promoters, leading to reinforcement and thereby robust regulation of gene expression. On the other hand, BC-G4s could bind different TFs in gene promoters and the first introns, allowing for independent modulation of transcription. To address this question, we calculated the proportion of TFs that bind both promoter and first intron G4s relative to the total TFs bound by first intron G4s, assessed on a gene-by-gene basis (Figure 4C). Our analysis revealed that 74% of first intron G4s share no TF with those in the cognate promoters, while approximately 4% share 10% of TFs with their respective promoter G4s. Thus, there is generally little overlap between G4–TF interactions in the first introns and their corresponding promoters, which infers that G4s within different regions could cooperatively regulate gene transcription in an independent manner (Figure 4C). Collectively, BC-G4s in promoters and the first introns of DEGs mostly contribute to additive modulation of gene expression by recruiting different TFs.

### 2.4. Promoter BC-G4s of DEGs Function as Hubs for TF Co-Binding

**Figure 5:**
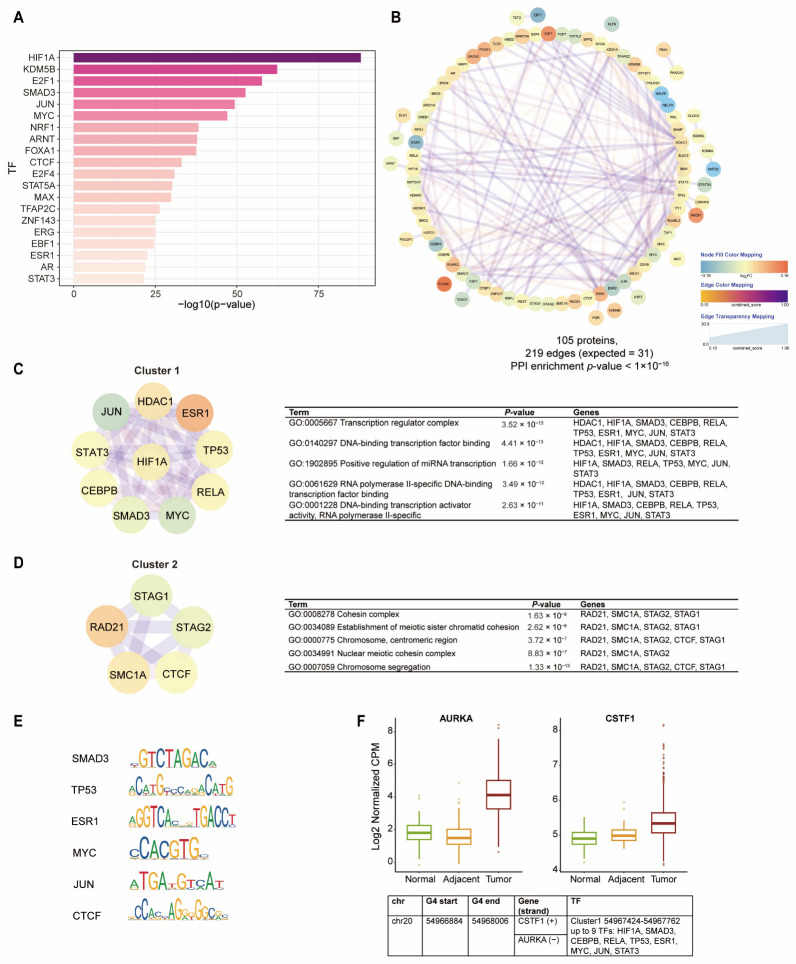
Transcription factor regulatory network analysis. (**A**) The enrichment barplot shows the top 20 TFs most significantly enriched in promoter BC-G4s of DEGs. (**B**) STRING analysis of protein–protein interaction of 105 proteins. A total of 219 edges are found between 86 of the proteins. Only 31 are expected by chance (*p*-value < 1 × 10^−16^). (**C**,**D**) show the top 2 significant protein clusters from the PPI network. (**C**) Cluster 1 and the top 5 enriched GO terms of cluster 1. (**D**) Cluster 2 and the top 5 enriched GO terms of cluster 2. (**E**) DNA-binding profiles retrieved from the JASPAR database for the 6 human stripe factors in two clusters except RAD21. (**F**) Boxplot of the expression levels of *AURKA* and *CSTF1*, two genes of which the promoter BC-G4 was enriched with the most TF–TF interactions. The significant difference is observed between tumor and NAT samples in these two genes.

Extensive research has manifested that the assembly of the general transcription factors on promoter DNA is required to facilitate transcription initiation [36,37]. Considering that BC-G4s in promoters bind far more TFs than those in the first introns, we specially focused on the possible implications of interactions between promoter BC-G4s and various TFs. We identified 105 proteins (mostly TFs and cofactors) abundant in DEG promoter BC-G4s [38], and the top 20 most significantly enriched TFs are shown in Figure 5A. In addition, 6 of the 20 TFs were up-regulated in tumor relative to NAT samples (Supplementary Figure S3). In concurrence with our previous observations, these TFs are largely connected with the cell cycle control such as E2F1 and E2F4. We further explored the collaborative patterns of TFs enriched in DEG promoter BC-G4s. The protein–protein interaction (PPI) of the set of 105 proteins was analyzed using the STRING database and portrayed as a network using Cytoscape (Figure 5B). PPI analysis highlighted a pronounced enrichment of confirmed interactions among these proteins (STRING PPI enrichment *p*-value < 1 × 10^−16^) which implies that these proteins may be biologically connected or function as a group. To focus on the key module in large-scale PPI network, significant protein clusters were identified subsequently using the Molecular Complex Detection (MCODE) plugin of Cytoscape which is a density-based graph theoretic clustering algorithm. We selected the top two clusters (including TFs and TF partners) with the highest scores which are cluster 1 composed of 10 nodes and 41 edges (HDAC1, HIF1A, SMAD3, CEBPB, RELA, TP53, ESR1, MYC, JUN, and STAT3) and cluster 2 composed of five nodes and nine edges (RAD21, SMC1A, STAG2, CTCF, and STAG1). Gene ontology (GO) enrichment analysis revealed proteins in cluster 1 belong to transcription regulator complex whereas proteins in cluster 2 are mainly implicated in establishment of meiotic sister chromatid cohesion (Figure 5C,D).

Furthermore, we were curious about whether promoter BC-G4s might be the main contributor to the co-binding patterns of TF clusters. To test this hypothesis, we extracted the overlaps among the DNA binding sites within each cluster using breast cancer ChIP-seq data (ChIP-ATLAS). We subsequently calculated the fold enrichment of these sites, over random chance, in the promoter BC-G4 regions of DEGs. Intriguingly, the co-localized sites of TFs exhibited marked abundance in promoter BC-G4s (Table 1). For instance, the sites with more than two co-localized TFs from cluster 1 overlap with 267 of the 424 G4 regions, which show significant enrichment at these promoter BC-G4s. Notably, most G4 regions of the DEGs are potentially bound by multiple TFs, implying that these promoter BC-G4s might act as central regulators for TF cooperation.

Recent research on TF binding profiles uncovered human “stripe factors” that frequently colocalize with various other TFs, creating vertical stripes in motif or ChIP-seq hierarchical clustering maps [39]. According to the published research, SMAD3, TP53, ESR1, MYC, and JUN from cluster 1, along with RAD21 and CTCF from cluster 2 are predicted to be “stripe factors”. It has been verified that the binding of these factors in human cells facilitates chromatin accessibility and recruits TF partners across the genome [39]. Additionally, their GC-rich binding motifs provide robust evidence for direct interactions with G4s which reinforces the notion that promoter BC-G4s are fundamental to the collaborative patterns of TFs (Figure 5E).

Based on the co-binding patterns of G4–TF and TF–TF interactions, we screened two genes of which the promoter BC-G4 is enriched with the most TF–TF interactions: the cell cycle-related gene *AURKA* and mRNA processing gene *CSTF1*, as candidates with the potential for future targeted therapy (Figure 5F). Specifically, these two genes both exhibit significantly elevated expression in tumor samples relative to NAT or normal samples. It has also been reported that overexpression of *AURKA* and *CSTF1* are associated with detrimental prognosis of breast cancer patients [40].

### 2.5. SNVs in BC-G4s Modulate G4 Structures and the Transcription of Breast Cancer-Associated Genes

A single nucleotide variation (SNV) within a G4 motif may cause a drastic change in G4 structure and, consequently, the expression of the regulated gene. Therefore, we investigated the impact of genome-wide G4–SNV interactions in breast cancer on the folding propensity of G4s and gene activity. To target SNVs that might influence the stability of G4s, we analyzed those located in G4 motifs within the BC-G4 peak regions. We downloaded the simple somatic mutation files of five breast cancer projects from the International Cancer Genome Consortium (ICGC) database release 28 and extracted a total of 4,103,395 SNV coordinates related to breast cancer. Among these, 3587 distinct SNVs were identified within 3523 motifs in BC-G4s, accounting for 3.7% of all BC-G4 motifs. The SNV-type distributions in BC-G4s were first computed in which we observed a slightly greater abundance of SNVs in G-tract regions when compared to loop regions and a high level of G > A mutation in G-tract regions (Supplementary Figure S4A).

**Figure 6:**
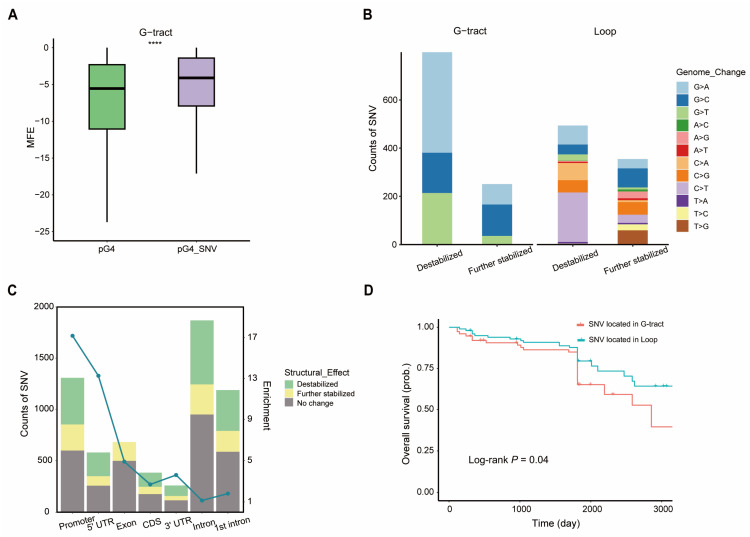
Analysis of SNVs associated with BC-G4s. (**A**) DNA-based minimum free energy (MFE) shows the thermodynamic changes caused by SNVs within the G-tract region of BC-G4s. (**B**) The distribution patterns of different SNV types in the G-tract or loop region leading to structural alterations of BC-G4s. (**C**) Barplots show the structural effects of G4-related SNVs in distinct genomic features (promoters, 5′ UTR, exons, CDS, 3′ UTR, introns, and the first introns). The green line depicts the enrichment of the SNV distribution within BC-G4s in comparison to the background SNV distribution in breast cancer. (**D**) Kaplan–Meier survival plot of SNV located in the G-tract and loop region. **** *p* < 0.0001, Wilcoxon two-sided test.

To probe into the effects of SNVs on BC-G4 structural stability, the minimum free energy (MFE) of BC-G4s with or without SNVs was calculated using the RNAfold program from the Vienna RNA package which is a powerful tool to predict secondary structures of single stranded RNA or DNA. In particular, the MFE of BC-G4s with SNVs in the G-tract region was found to be significantly higher than the MFE of the original sequences (Figure 6A; Wilcoxon two-sided test, *p*-value < 2 × 10^−16^) whereas this phenomenon was not observed in the loop region (Supplementary Figure S4B). The result suggests that SNVs within the G-tract regions lead to a pronounced increase in structural instability.

We further analyzed the SNV types affecting BC-G4 structural stability and corroborated that SNVs disrupting G-tract regions are more prone to induce BC-G4 structural alterations, predominantly leading to G4 destabilization as expected. In addition, specific transition events, such as G > A in G-tract regions and C > T in loop regions, are more susceptible to triggering structural destabilization of BC-G4s (Figure 6B). Conversely, a small minority of SNVs further stabilize G4 structures, potentially due to the formation of Watson–Crick base-pairs induced by the SNVs within G4 structures [41].

In our research about the biomedical importance of SNVs within BC-G4s concerning gene regulation, the genomic annotation of SNVs revealed a notable enrichment in gene introns and promoter regions. In particular, intronic SNVs show more prevalence in the first introns. After being normalized by the number of SNVs in the background, i.e., both inside and outside of the G4 motifs, G4-related SNVs exhibit noteworthy abundance in the promoter and 5′ UTR regions (Figure 6C; Fisher’s exact test *p*-value < 2.2 × 10^−16^ for both promoter and 5′ UTR regions). These results are indicative of stronger purifying selection acting on G4 loci for motif retention than on the remaining sequences in genic functional regions, thus enabling the stability and function of BC-G4s [42]. To figure out the clinical significance of SNVs, the survival analysis of SNV location was performed using the data from ICGC which demonstrated that breast cancer patients with SNVs in G-tract regions have a lower overall survival probability than those with SNVs in loop regions (Figure 6D; log-rank test, *p*-value = 0.04).

**Figure 7:**
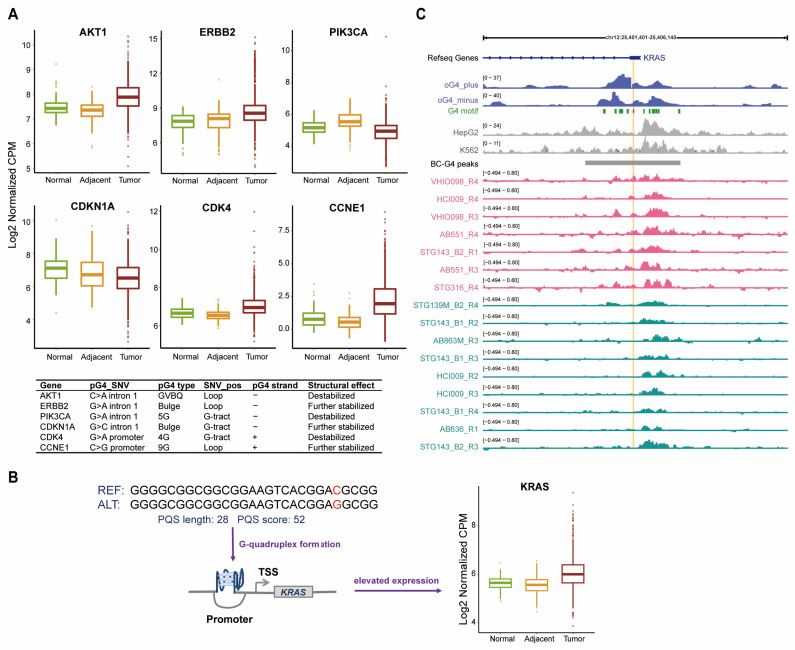
The vital genes and SNVs associated with BC-G4s. (**A**) Boxplots of the expression levels and the corresponding SNV information of the top 6 seed genes in which SNVs in gene promoters and the first introns cause structural alterations of BC-G4s. (**B**) Boxplots of *KRAS* expression and the formation of a new G4 motif in the promoter region. (**C**) Track intensities of G4-seq for observed G4 (oG4) in vitro (blue), G4 ChIP-seq for HepG2 and K562 cell lines (light gray), qG4-ChIP-seq for examples of several PDTX models (pink and green) around the SNV position (red) in the *KRAS* promoter region.

In view of the crucial importance of BC-G4s in gene promoters and the first introns, we selected the genes accommodating SNVs that affect stability of these BC-G4s. We next input these genes to Phenolyzer, a tool for prioritizing human disease genes based on existing knowledge, to identify those associated with breast cancer [43]. From the top 10 genes with the highest scores, we selected 6 seed genes to examine their expression levels across tumor, NAT, and normal conditions (Supplementary Table S2). A seed gene refers to the gene with direct relations to breast cancer, based on the existing databases. Notably, *AKT1*, *ERBB2* (*HER2*), *CDK4*, and *CCNE1* are significantly amplified in tumor samples while *PIK3CA* displays the lower expression level in breast cancer (Figure 7A). According to previous research, each SNV may exert distinct effects depending on a combination of multiple factors and our findings further proved this observation [27]. *AKT1* and *PIK3CA* are both implicated in PI3K/AKT pathway which is vital to cell growth and overexpression of *ERBB2* also contributes to cell proliferation. The other three genes including *CDKN1A*, *CDK4*, and *CCNE1* are all involved in cell cycle control.

Moreover, we focused on the gain of G4 motifs induced by SNVs. We first obtained 30 nucleotides from both sides of each SNV and identified pG4 patterns on both strands in the reference (REF) and alternative (ALT) sequences, respectively, using the pqsfinder package. The pqsfinder result manifested that 4613 SNVs lead to the formation of new G4 motifs. We subsequently overlapped these regions with experimentally validated G4 peaks and found 272 newly formed G4 motifs in breast cancer. Strikingly, a new G4 motif is formed in the promoter region of *KRAS* which exhibits a markedly increased expression level in breast cancer (Figure 7B).

In addition, we examined whether SNV-driven G4 signals in the promoter region of *KRAS* could be detected in breast cancer or other cellular contexts to confirm cancer-type specific G4 formation. Intriguingly, the relatively weak G4 signals observed around this site, generated in vitro or in vivo from HepG2 and K562 cell lines, indicated challenges in forming a stable G4 structure in these cells (Figure 7C). However, a part of the breast cancer samples exhibited pronounced signals at the SNV while others did not (Figure 7C). In summary, these results imply that this SNV in breast cancer may be fundamental to the formation of a new *KRAS* G4 structure.

## 3. Discussion

In this work, we have conducted a comprehensive and in-depth research on genome-wide characteristics of BC-G4s generated from public G4 data in breast cancer. Our results reveal that BC-G4s play a prominent part in the regulation of gene expression and cell cycle pathway. We also notice that BC-G4s in the first introns have more TF interactions than downstream introns. Based on TF enrichment patterns and the impact of SNVs on BC-G4s, we have identified several important G4s and G4-related genes that may serve as the potential drug target for future breast cancer therapy.

Advances in computational methods for pG4 prediction and G4 sequencing technologies have facilitated the investigation of G4 structures in different cell lines [44,45,46]. We have identified BC-G4s derived from PDTX samples and MCF7 cell line, thus including multiple subtypes in breast cancer. Previous works have emphasized the specificity of differentially expressed G4 regions in distinct breast cancer subtypes, while we aimed to provide a comprehensive overview of all possible emerging BC-G4s throughout the human genome. For this purpose, we surveyed their presence in gene regulatory elements. The prevalence of BC-G4s in promoters and introns aroused our interest in the exploration of the correlation between genes marked by promoter or intronic BC-G4s and their expression levels. Remarkably, we demonstrated the positive effects of BC-G4s in gene promoters and the first introns on elevated gene expression (Figure 2B,F), which can be attributed to the proximity of these regions to the TSSs.

**Figure 8:**
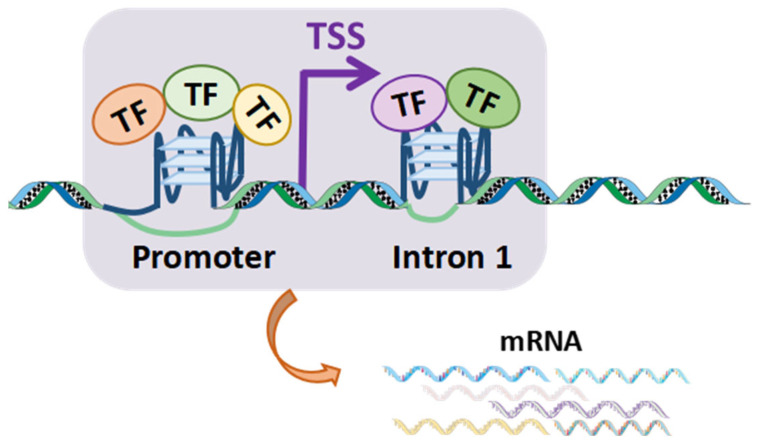
TF interactions with BC-G4s in promoters and the first introns cooperatively regulate gene transcription (BC-G4s form on the non-transcribed strand).

A prior study correlated enhanced gene transcription with various TFs binding to promoter G4s [17]. Herein, we extend this pattern to BC-G4s in the first introns. We observed that BC-G4s in the first introns could also recruit TFs and facilitate gene expression, implying a universally positive role of G4s located both upstream and downstream of the TSSs in transcription. Notably, TF interactions with BC-G4s in the first introns are not redundant with those in promoters, suggesting cooperative regulation of gene expression (Figure 8). A pioneer analysis of the interactions between TFs and the first introns in *Caenorhabditis elegans* elaborated the combined effects of multiple regulatory regions [22]. We further confirmed that G4s may be fundamental to the additive regulation of gene expression.

Replicative immortality is one of the hallmarks of cancer that typically arises from the deregulation of cell cycle pathways. The disruption of cell cycle control caused by molecular alterations in breast cancer drives genome instability and tumor progression [47]. The cell cycle-targeted therapy has been considered a promising anti-cancer strategy. We observed that BC-G4s were apparently implicated in the modulation of cell cycle pathways which inspired us to investigate novel inhibitors of cell cycle regulators from the perspective of BC-G4s (Figure 3B,C). We have selected several genes probably regulated by BC-G4s including *CDK4*, *CCNE1*, *CDKN1A*, and *AURKA* which are components of the cell cycle machinery with clinical potential. The others such as *AKT1*, *PI3KCA*, and *ERBB2* are also closely related to cell cycle regulation. More specifically, upstream oncogenic signaling such as PI3K/AKT/mTOR signaling pathway leads to the activation of cyclin D-CDK4/6 complex, which facilitate the phosphorylation of RB1 further contributing to E2F release and transition of cell cycle from G1 to S phase [48,49,50]. Notably, E2F proteins like E2F1 and E2F4 revealed a marked enrichment in promoter BC-G4s of DEGs (Figure 5A). Over the past two decades, inhibitors of CDK4/6 have been widely used as first-line therapy for hormone receptor-positive and human epidermal growth factor receptor 2 (HER2)-negative metastatic breast cancer [51,52,53,54,55,56]. However, cell cycle alterations as well as upstream oncogenic signal transduction alterations can promote resistance to CDK4/6 inhibitors [57]. Given these findings, combinatorial strategies could be a great aid and advantage in conquering the problems and targeting the BC-G4s involved in cell cycle processes hopefully represents a breakthrough in clinical trials [48]. Furthermore, the BC-G4 in the promoter of *AURKA* is enriched with multiple TF–TF interactions suggesting the vigorous activity of this G4 region in breast cancer (Figure 5F). In recent years, the emergence of more effective biochemical methods has greatly contributed to the study of G4-protein interactions in diseases, thus adding new dimensions to clinical applications [58,59].

The well-known proto-oncogene *KRAS* is one of the most frequently mutated genes in various cancers and the overexpression of *KRAS* can result in poor survival. The core promoter region of human *KRAS* extending from +50 to −510 bp in relation to the TSS is characterized by a high G/C content [60]. *KRAS*-G4 comprising a GC-rich nuclease hypersensitive element (NHE) has been shown to be a transcriptional modulator recognized by several nuclear proteins [61,62,63]. Intriguingly, we discovered that the promoter region of *KRAS* probably formed a novel G4 proximal to the TSS based on the occurrence of SNVs in breast cancer which is distinct from the well-established *KRAS*-G4 previously. Remarkably, the first approval of targeted therapy for non-small cell lung carcinoma (NSCLC) patients with *KRAS* mutation has shed light on the development of *KRAS*-targeting drugs [64]. Additionally, the structural insights into the *KRAS*-G4-ligand interactions contribute to the rational design of *KRAS*-G4 specific drugs [62]. Although we are not clear about how this newly formed G4 induced by SNV correlates with the overexpression of *KRAS*, it will pave the way for drug innovation in treatment of *KRAS*-mutant breast cancer patients.

Taken together, we emphasize that the presence of BC-G4s in promoters and the first introns is inextricably linked with enhanced gene expression and have a pivotal role in TF binding. Notably, it has been proved that BC-G4s may cover multiple aspects of cell cycle regulation. In addition to the structural alterations caused by SNVs, we also observed the formation of a novel G4 motif in the promoter region of *KRAS* in breast cancer. Collectively, studies on G4-protein interactions and G4-variant effects will present a promising new strategy for drug design in breast cancer.

## 4. Materials and Methods

### 4.1. G4 Dataset Acquisition

In this study, we mainly focused on the experimentally validated G4 dataset in breast cancer. First, we downloaded the G4 coordinate files of 22 breast cancer patient-derived tumor xenograft (PDTX) models and MCF7 breast adenocarcinoma cell line from the Gene Expression Omnibus (GEO) database (GSE152216 and GSE181373) that were obtained from quantitative G4-chromatin immunoprecipitation (ChIP)-seq and single-nuclei (sn) CUT&Tag both using G4 structure-specific antibody BG4 [29,31]. Then we merged all the G4 peaks from breast cancer tissues and cell line to generate a single G4 DNA consensus of 47,797 G4 regions (hereafter named as BC-G4s) using the bedtools merge function (version 2.29.1). Genomic feature distributions of BC-G4 were analyzed using the ChIPseeker (version 1.34.1) package [65].

The putative G-quadruplex (pG4) regions in the human genome hg19 assembly across both strands were obtained from GSE133379 [46], which includes multiple G4 motif subtypes such as 4G, 4GL15, Bulges, and GVBQ. The intersections between the putative and experimental G4 were acquired by using bedtools intersection function. The in vitro G4 data captured by the G4-seq technique were extracted from GSE63874 [44]. The G4 files for HepG2 and K562 cell lines derived from G4 ChIP-seq were downloaded from GSE145090 [17].

### 4.2. RNA-Seq Dataset Acquisition and Processing

We chose RNA-seq datasets from TCGA and GTEx since they are two consortia for population-level studies where samples are from patients or healthy individuals. The raw feature counts based on an identical processing protocol described in Rahman et al. [33] were obtained from GSE62944 (1119 tumor samples and 113 NAT samples in breast cancer) and GSE86354 (92 normal samples from GTEx in breast tissue) (Supplementary Figure S2A) [33,34]. Dimensionality reduction was performed through the Rtsne (version 0.17) package on the log2 CPM (counts per million) values calculated by the edgeR (version 3.40.2) package (Supplementary Figure S2B). Since large-scale RNA-seq datasets generated in different laboratories and at different times contain batch-specific systematic variations, we employed the RUVg method from the RUVseq package (version 1.32.0) for batch-effect removal and data integration [35]. The list of housekeeping genes was considered as a good set of negative controls suggested by the official user guide [66]. Batch effect correction could be visualized in the relative log expression (RLE) plot where the distributions were centered around the zero line and undistinguishable between the conditions (Supplementary Figure S2C).

### 4.3. Differential Expression Analysis

The count matrix processing was performed by edgeR package [67]. We filtered out genes with very low counts followed by TMM (the trimmed mean of M values) normalization and then transferred counts to CPM. To avoid exaggerated false positives for large-sample-size data, the Wilcoxon rank-sum test rather than parametric methods was selected to identify differentially expressed genes (DEGs) for its solid false discover rate (FDR) control and good power [68]. Differential expression analysis was performed between pairs of the three conditions (Supplementary Table S1). Each gene’s CPM was input into the wilcox.test function in R for *p*-value calculation. The FDR value was obtained using the Benjamini and Hochberg method. DEGs between tumor and NAT samples was identified if (1) FDR < 0.01, and (2) ≥4-fold expression change. KEGG pathway enrichment plot was created using the clusterProfiler (version 4.6.2) package.

### 4.4. BC-G4 Overlapping Gene Promoter and Intron Expression Analysis

Promoter (1 kb upstream from the transcription start site, TSS) and intron coordinates were generated using the hg19 assembly (https://www.gencodegenes.org/human/release_19.html (accessed on 1 July 2013)). The gene promoters and introns harboring BC-G4 were obtained using the bedtools annotate function. Significance was tested using the Wilcoxon test.

### 4.5. Transcription Factor Enrichment Analysis

The TFs enriched in promoter and intronic BC-G4s of DEGs were calculated using the ChIP-Atlas Enrichment Analysis web tool (https://chip-atlas.org/enrichment_analysis (accessed on 16 May 2024)) with the following parameters [69,70]: Experiment type: ChIP: TFs and others; Cell type Class: Breast; Threshold for Significance: 500; Enter dataset A: promoter and intronic BC-G4s of DEGs in bed format; Enter dataset B: Random permutation of dataset A (100 permutation times). The result table containing 11 tab-separated columns was downloaded. Rows were filtered based on multiple criteria: no. 5 cell belongs to breast cancer cells; no. 9 log(*p*-value) < −3 and no. 11 fold enrichment > 1. 148 TFs in total were determined for further study.

### 4.6. ChIP Binding Site Analysis

ChIP-seq data of TFs in breast cancer were downloaded from http://chip-atlas.org/peak_browser (accessed on 16 May 2024). The midpoint of the ChIP-seq BED region was defined as the binding site. Promoter and intronic BC-G4s of DEGs were analyzed individually to verify whether the TF ChIP peak midpoints were located between the start and end of the fragment.

### 4.7. Transcription Factor Network Analysis

The protein–protein interaction (PPI) network of 105 TFs enriched in promoter BC-G4s of DEGs with the cutoff interaction score set at 0.9 was constructed using the Search Tool for the Retrieval of Interacting Genes/Proteins (STRING) website (http://string-db.org/) [71]. The network was subsequently portrayed using Cytoscape software (version 3.10.0) [72]. The PPI enrichment *p*-value was sourced from the STRING web tool. Next, the central clustering modules in the PPI network were screened out using the “MCODE” plugin of Cytoscape with the default parameters (Network Scoring: Include Loops: false; Degree Cutoff: 2; Cluster Finding: Node Score Cutoff: 0.2; Haircut: true; Fluff: false; K-Core: 2; Max. Depth from Seed: 100) and top 2 clusters with the highest scores were selected. ChIP-seq BED files of 15 TFs from the top 2 clusters were downloaded from ChIP-Atlas Peak Browser web tool. The peaks were further screened based on the IDs that were previously determined in the TF enrichment result table. The common intervals among multiple BED files from the same cluster were calculated using bedtools multiinter. The enrichment analysis of intersections among TF binding sites in promoter BC-G4s of DEGs was conducted following the same steps as TF enrichment analysis. *p*-values were calculated with two-tailed Fisher’s exact probability test. TF binding motifs in humans were downloaded from JASPAR (https://jaspar.elixir.no/) [73].

### 4.8. SNV Dataset Acquisition

The International Cancer Genome Consortium (ICGC) has been extensively used as a tool for cancer genomic analyses and data generated from the TCGA was included in its current release (ICGC release 28, https://dcc.icgc.org/releases/release_28 (accessed on 26 November 2019)) [74]. ICGC release 28 contains five breast cancer projects including (1) BRCA-EU Breast ER+ and HER2− Cancer—EU/UK, (2) BRCA-FR Breast Cancer—FR, (3) BRCA-KR Breast Cancer—Very young women—KR, (4) BRCA-UK Breast Triple Negative/Lobular Cancer—UK and (5) BRCA-US Breast Cancer—TCGA, US. Single nucleotide variation (SNV) coordinates in breast cancer were extracted from simple somatic mutation files downloaded from these five projects. We removed apparently duplicated SNVs and obtained 4,103,395 SNV coordinates in total for subsequent analyses.

The donor survival time data were downloaded from https://icgc-xena-hub.s3.us-east-1.amazonaws.com/download/donor%2Fdonor.all_projects.overallSurvival.gz (accessed on 26 November 2019).

### 4.9. SNV Affecting BC-G4s

Overlaps between SNVs and BC-G4 motifs were calculated using the bedtools intersect function. To evaluate the effect of SNVs on the structural stability of BC-G4s, we compared the minimum free energy (MFE) of BC-G4s before and after the occurrence of SNV computed by the Vienna Package RNAfold (version 2.6.4) [75]. A lower MFE value indicates a more stable structure while a higher MFE value means a less stable structure. G4-related SNVs in genomic regions were annotated using bedtools and the enrichment score for each annotation was calculated by taking the formula described as follows, by comparing the proportion of G4-related SNVs in a specific genomic region to that of background SNVs in breast cancer:

$${Enrichment\;Score}_{G4,\;region}=\frac{N_{G4,\;region}/N_{G4,\;total}}{N_{BC,\;region}/N_{BC,\;total}}$$

where *N_G4,region_* denotes the number of G4-related SNVs in the given genomic region. *N_G4,total_* represents the total number of G4-related SNVs. *N_BC,region_* is the number of breast cancer SNVs in the genomic region. *N_BC,total_* stands for the total number of breast cancer SNVs. An enrichment score greater than 1 suggests an over-representation of G4-related SNVs in the specified genomic region relative to the background SNV distribution in breast cancer. Fisher’s exact tests were conducted to assess the enrichment of the SNVs in BC-G4s.

In addition, 30 nucleotide sequences flanking the SNV position were extracted by bedtools getfasta. The reference and variant sequence in each pair were both searched for the putative quadruplex motif using the pqsfinder (version 2.14.1) package with default parameters [76]. We selected the newly formed G4 motifs with in vivo experimental support. Based on our previous study, genes harboring SNVs located in the first introns and upstream regions that impact G4 structures or facilitate to form new G4 motifs were input into Phenolyzer to search for the seed genes associated with breast cancer (Supplementary Table S2, Supplementary Figure S4C,D) [43]. The Kaplan–Meier method was used to estimate overall survival (OS), and the log-rank test was utilized to compare them.

### 4.10. Statistical Analysis

Wilcoxon tests were conducted to compare gene expression values between groups in this study. To validate the higher frequency of BC-G4s in the first introns, a permutation test was performed by randomizing the intronic BC-G4 positions across all intron regions using bedtools shuffle function. The proportion of BC-G4s in the first introns was calculated in each of 10,000 random permutations and compared to the observed proportion. We conducted Fisher’s exact test to assess the enrichment of BC-G4s in promoters and the first introns of DEGs. Spearman’s correlation coefficient was calculated to investigate the relationship between G4–TF interactions in the first introns and the cognate promoters.

## 5. Conclusions

In summary, this work is a pioneer exploration of genome-wide characteristics of BC-G4s. Based on TF enrichment patterns and the impact of SNVs on BC-G4s, we have identified several important G4s and G4-related genes that may serve as the potential drug targets for future breast cancer therapy. It is an appealing prospect that our analytical framework could also be useful in the search for novel cancer treatments beyond breast cancer.

## Figures and Tables

**Table 1:** The co-localized sites of TFs are significantly enriched in promoter BC-G4s of DEGs (G4Rs), and the sites are classified by the number (#) of these binding proteins.

Class Labeled by # of Proteins	# of Sites	Overlaps in G4R	Overlaps in Control	*p*-Value
Cluster 1				
≥2	56,089	267/424	6/424	2.00578 × 10^−97^
≥3	27,651	217/424	3/424	1.801687 × 10^−76^
≥4	16,064	172/424	3/424	2.081164 × 10^−56^
≥5	9648	116/424	3/424	1.390859 × 10^−34^
≥6	3483	44/424	0/424	3.505748 × 10^−14^
≥7	739	15/424	0/424	5.381052 × 10^−5^
≥8	127	5/424	0/424	0.06176341
Cluster 2				
≥2	47,269	164/424	10/424	1.053576 × 10^−44^
≥3	32,097	74/424	5/424	3.748428 × 10^−18^
≥4	23,600	42/424	3/424	3.429332 × 10^−10^
≥5	18,483	36/424	1/424	2.652893 × 10^−10^

## Data Availability

The BC-G4 data that support the findings are openly available in github at https://github.com/hlshu/BC-G4peak/ (accessed on 11 June 2025).

## Supplementary Materials

The following supporting information can be downloaded at: https://www.mdpi.com/article/10.3390/ijms26146874/s1.

## Abbreviations

The following abbreviations are used in this manuscript:

G4	G-quadruplexes
TSS	transcription start site
TF	transcription factor
SNV	single nucleotide variant
PDTX	patient-derived tumor xenograft
DEG	differentially expressed gene
PPI	protein–protein interaction
MFE	minimum free energy
