# Supplementary material for: The Relevance of G-Quadruplexes in Gene Promoters and the First Introns Associated with Transcriptional Regulation in Breast Cancer
Source: Int J Mol Sci. 2025 Jul 17;26(14):6874. doi: 10.3390/ijms26146874 (PMC12295768; doi:10.3390/ijms26146874)
Supplement: Supplementary file 1 [file ijms-26-06874-s001.zip › ijms-3721861 Supplementary/Supplementary.pdf]

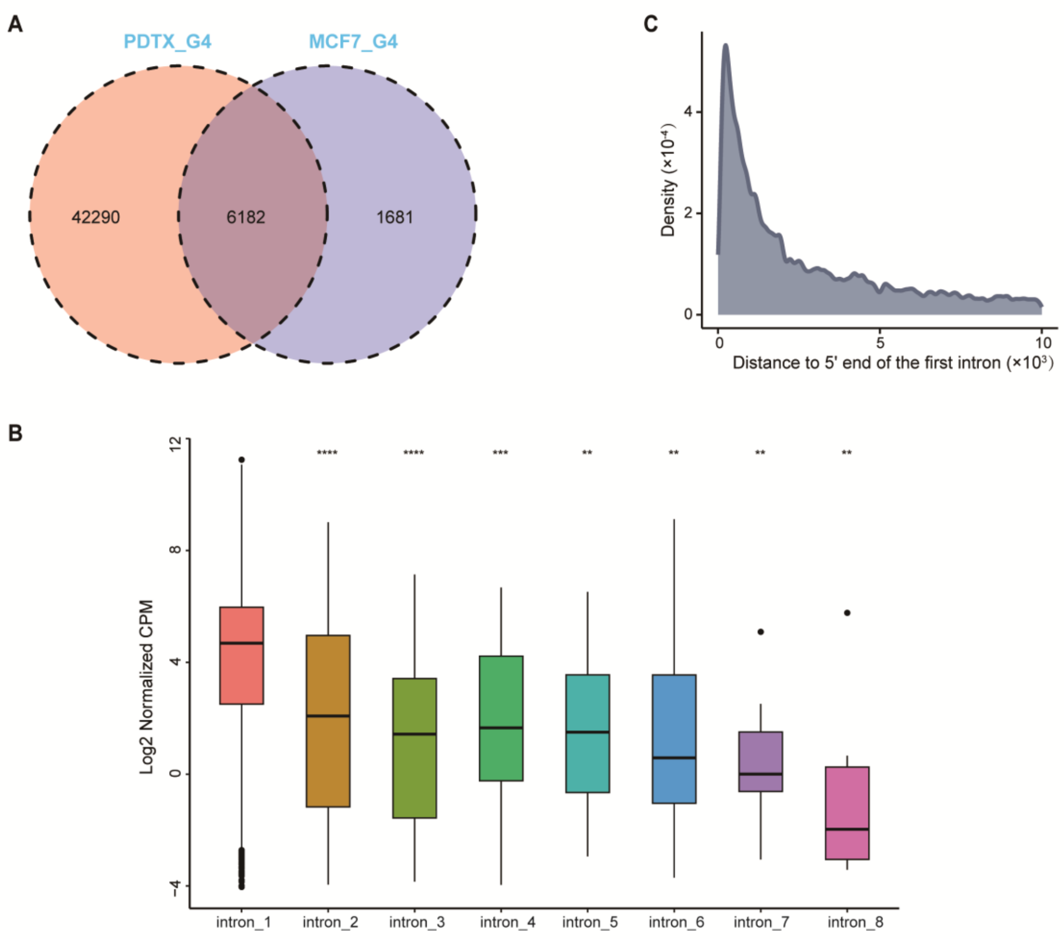

Supplementary Figure S1. Characteristics of BC-G4s. **(A)** Venn diagram of G4 data from PDTX models and MCF7 cell lines. **(B)** Boxplots show the expression levels of genes with BC-G4s in the first introns compared with those harboring G4s in the other introns. **(C)** Density plot illustrates the distance of BC-G4s to 5' end of the first intron. ** $P < 0.01$, *** $P < 0.001$, **** $P < 0.0001$, Wilcoxon test.

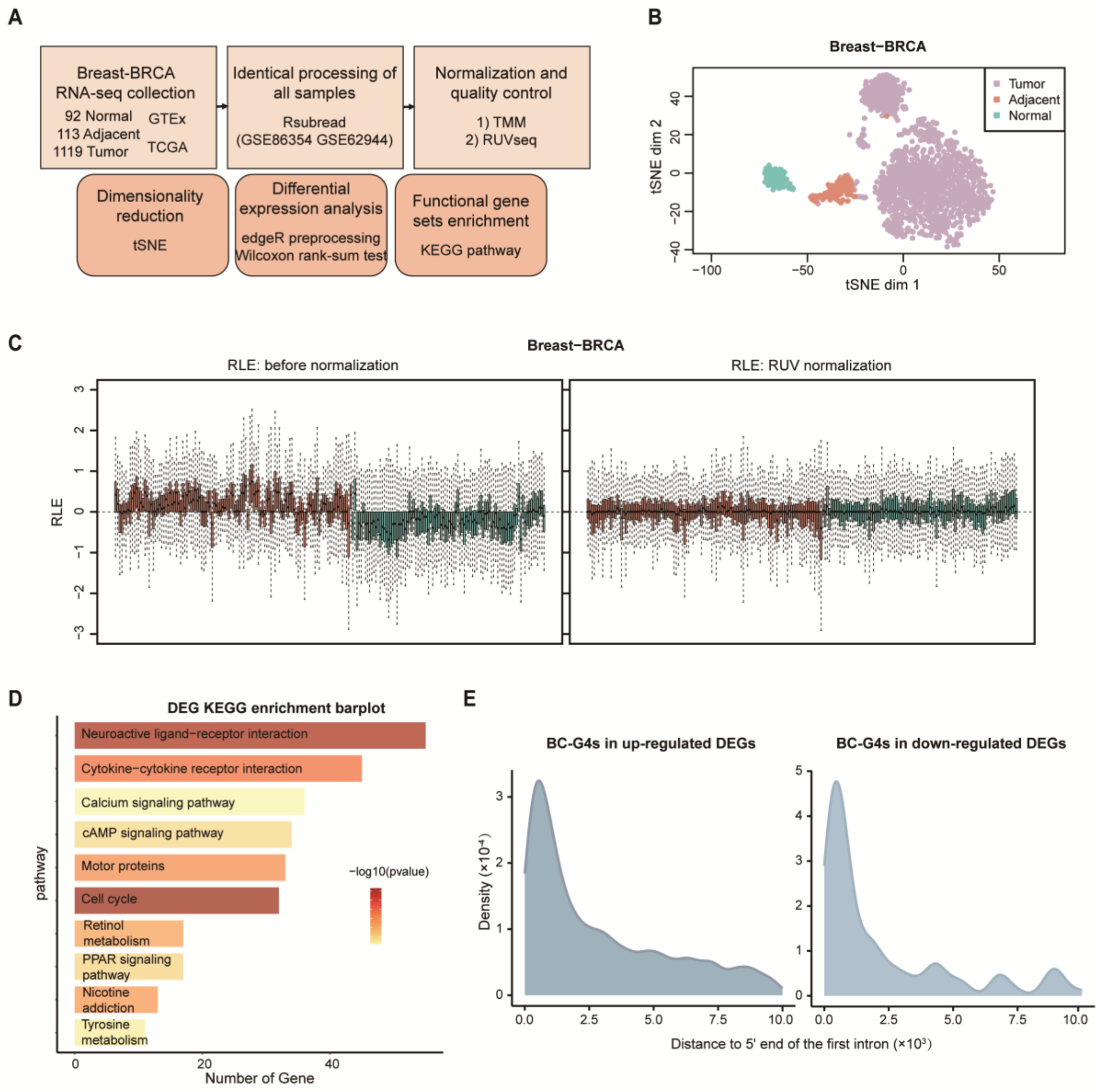

Supplementary Figure S2. Differential expression analysis in breast cancer. **(A)** Study design of differential expression analysis. We collected raw count data of 1119 tumor, 112 tumor-adjacent normal (NAT) samples in breast cancer and 92 normal samples in breast tissues. We then utilized several methods to identify and analyze the differentially expressed genes. **(B)** Dimentionality reduction of TCGA and GTEx samples. t-SNE plot for three conditions shows the NAT samples (orange) are in between the normal (green) and tumor (purple) samples. **(C)** Stringent removal of batch effects using RUVseq package. The relative log expression (RLE) plot shows results using RUV normalization. Without RUV normalization using housekeeping genes, there are apparent differences in RLE between TCGA NAT samples (orange) and GTEx normal samples (green), after RUV the RLE is centered around zero. **(D)** KEGG pathway enrichment patterns of DEGs in breast cancer. **(E)** Density plots show the distance of BC-G4s to 5' end of the first intron in up-regulated and down-regulated DEGs.

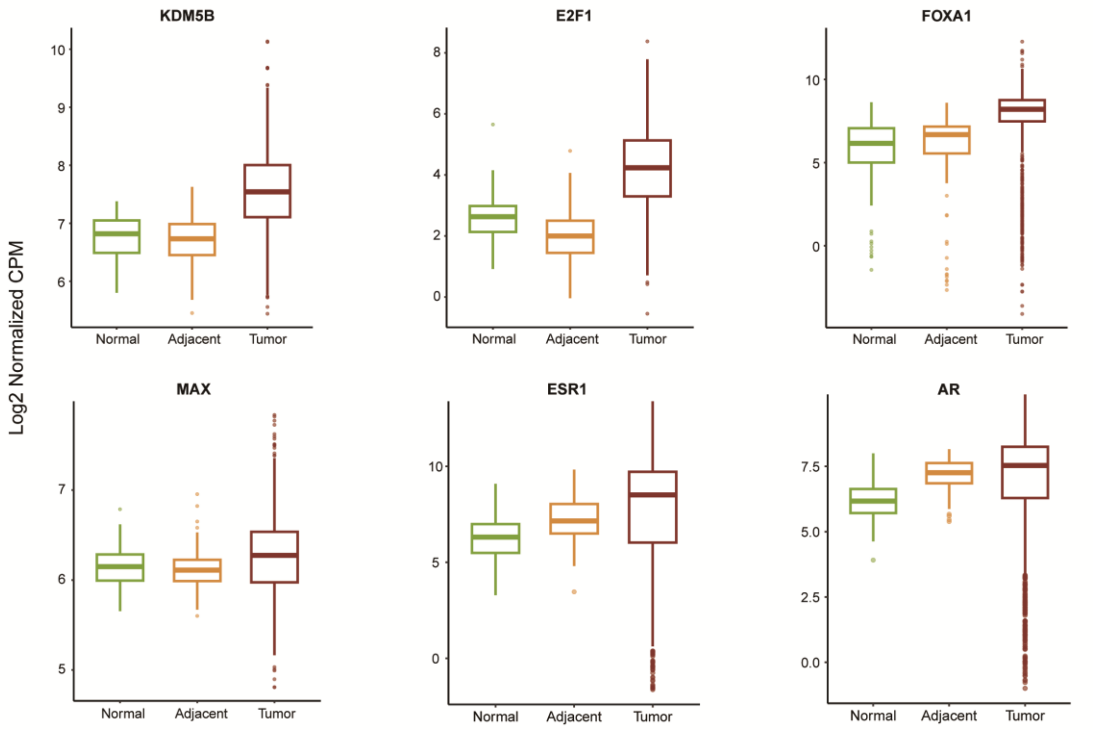

Supplementary Figure S3. The gene expression boxplots of up-regulated transcription factors in tumor relative to NAT samples.

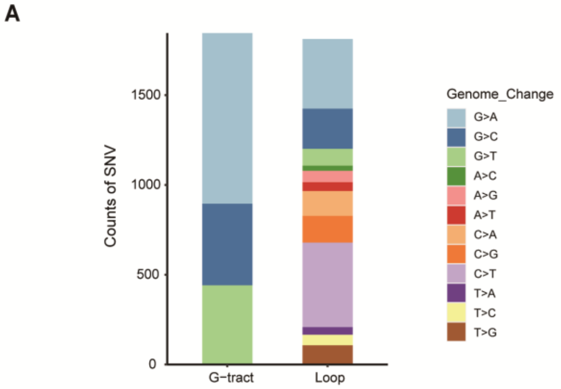

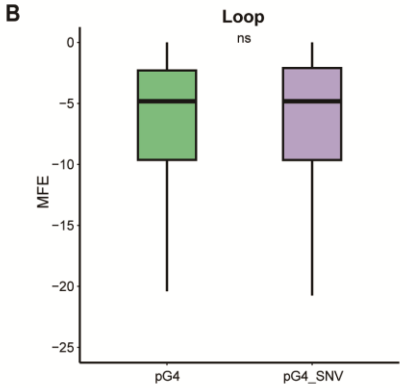

C

Rank	Gene	ID	Score	Status	Haploinsufficiency Score	Gene Intolerance Score
1	AKT2	208	0.2455	Seed Gene	0.932	0.8364000944
2	KRAS	3845	0.237	Seed Gene	1.000	0.5712432177
3	RXRA	6256	0.2065	Predicted	0.743	0.8487850908
4	KLLN	100144748	0.1576	Seed Gene	0	0.09170795
5	SMAD7	4092	0.1459	Predicted	0.604	0

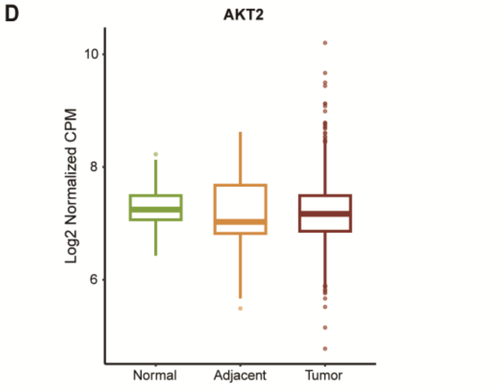

Gene	pG4_SNV	pG4	pG4 strand
AKT2	A>G intron 1	GCGGGACGAGGCGGGTCAGTTTAGATG GGTGCGGGGG	-

Supplementary Figure S4. Joint analysis of BC-G4s and SNVs. **(A)** The counts of different SNV types that occur in the G-tract or loop region of BC-G4s. **(B)** DNA-based minimum free energy (MFE) shows the thermodynamic changes caused by SNVs within the loop region of BC-G4s. **(C)** The top 5 genes where a new G4 motif forms in promoters or the first introns using the Phenolyzer tool. **(D)** Boxplots of AKT2 expression and the formation of a new G4 motif in its first intron. No significant difference in gene expression of AKT2 was observed between tumor and NAT samples

Supplementary Table S1. Differentially expressed gene list in breast cancer. **Sheet 1** Tumor relative to NAT samples. **Sheet 2** Tumor relative to normal samples. **Sheet 3** NAT relative to normal samples.

Supplementary Table S2. The top 10 genes in which SNVs in gene promoters and the first introns cause structural alterations of BC-G4s using the Phenolyzer tool.

Rank	Gene	ID	Score	Status	Haploinsufficiency Score	Gene Intolerance Score
1	AKT1	207	0.371	Seed Gene	0.997	0.91678461901
2	ERBB2	2064	0.2921	Seed Gene	1.000	0.8171738618
3	PIK3CA	5290	0.2778	Seed Gene	0.952	0.7580797358
4	CDKN1A	1026	0.2715	Seed Gene	0.980	0.2153809861
5	CDK4	1019	0.2596	Seed Gene	1.000	0.6738027837
6	PRKCA	5578	0.2376	Predicted	0.827	0.6929700401
7	CCNE1	898	0.2235	Seed Gene	1.000	0.7106628922
8	POLR2A	5430	0.215	Predicted	0.998	0.99681528662
9	PPP1CA	5499	0.211	Predicted	0.997	0.713670677
10	RXRA	6256	0.2065	Predicted	0.743	0.8487850908
